# Intratumoural Treatment of 18 Cytologically Diagnosed Canine High-Grade Mast Cell Tumours With Tigilanol Tiglate

**DOI:** 10.3389/fvets.2021.675804

**Published:** 2021-08-27

**Authors:** Graham K. Brown, Justine E. Campbell, Pamela D. Jones, Thomas R. De Ridder, Paul Reddell, Chad M. Johannes

**Affiliations:** ^1^QBiotics Group Limited, Yungaburra, QLD, Australia; ^2^Department of Clinical Sciences, Iowa State University College of Veterinary Medicine, Ames, IA, United States

**Keywords:** high-grade, mast cell tumour, mast cell tumor, tigilanol tiglate, intratumoural, intratumoral, canine (dog), cytological grading

## Abstract

Canine high-grade mast cell tumours (HGMCT) are associated with a poor prognosis, are inherently more invasive, and have higher rates of local recurrence. The primary aim of this retrospective study was to assess the efficacy of intratumoural tigilanol tiglate (TT) as a local treatment option. Eighteen dogs with mast cell tumours (MCT) cytologically diagnosed by veterinary pathologists as either high-grade or suspected high-grade MCT were treated with TT. The TT dose was based on tumour volume (0.5 mg TT/cm^3^ tumour volume) and delivered intratumourally using a Luer lock syringe and a fanning technique to maximise distribution throughout the tumour mass. Efficacy was assessed on the presence/absence of a complete response (CR) to therapy at days 28 and 84 using response evaluation criteria in solid tumours (RECIST). For dogs not achieving a CR after 28 days, the protocol was repeated with a second intratumoural TT injection. Ten out of 18 dogs (56%) in this study achieved and maintained a CR to at least 84 days after their first or second treatment. Six patients were alive and available for evaluation at 2 years, three of those were recurrence free, and a further three patients were recurrence free following a second treatment cycle. Tigilanol tiglate shows efficacy for local treatment of HGMCT, with higher efficacy noted with a second injection if a CR was not achieved following the first treatment. In the event of treatment site recurrence (TSR), the tumour may be controlled with additional treatment cycles. Tigilanol tiglate provides an alternative local treatment approach to dogs with HGMCT that would either pose an unacceptable anaesthetic risk or the tumour location provides a challenge when attempting surgical excision.

## Introduction

Mast cell tumours (MCT) are a common canine skin cancer accounting for up to 21% of all skin tumours (1–4). Common treatment options include surgery, radiation, and chemotherapy used alone or in combination based on tumour location, size, and prognostic factors including tumour grade and markers of cellular proliferation (1, 3–8). Using a 2-tier system, high-grade mast cell tumours (HGMCT), are reported in the literature to make up between 11 and 35% of diagnosed MCT (2, 9–11). High-grade mast cell tumours have a comparatively poor prognosis because of higher rates of local recurrence, regardless of resection with histologically tumour free margins, and a high propensity for metastasis to distant sites (2, 12). For most HGMCT patients, the recommended mainstay of treatment is complete surgical resection of the lesion combined with chemotherapy as a systemic adjunct or alternatively, prophylactic irradiation of local lymph nodes (2, 13–15). Where local control has been inadequate, radiation therapy may be used alone or in combination with chemotherapy for improved outcomes (1, 5, 6, 8, 12–14, 16–20). Lesion proximity to critical structures, patient co-morbidities, anaesthetic risk, and financial constraints are factors that may limit the application of these treatment modalities in some clinical situations (1, 4, 8, 21). In these situations, intratumoural therapy (which achieves high local drug concentrations of potent chemotherapeutics while minimising systemic toxicity) may provide a viable treatment option where surgical intervention alone is unlikely to be curative without additional therapy (22, 23).

Tigilanol tiglate (TT) is a novel small molecule approved by the European Medicines Agency (EMA), Swissmedic and United Kingdom Veterinary Medicines Directorate in January 2020, United States Food and Drug Administration (USFDA) in November 2020, and Australian Pesticides and Veterinary Medicines Authority (APVMA) in July 2021 as a local intratumoural treatment for canine MCTs (24–26). The approved indication varies between countries. All approved labels are indicated, regardless of grade, for the intratumoural treatment of non-metastatic cutaneous MCT anywhere on the body and non-metastatic subcutaneous MCT at or below the elbow or hock. However, the EMA confines treatment to “non-resectable” tumours where the USFDA and APVMA does not restrict use to non-resectable. Finally, the USFDA and APVMA labels are less restrictive with regard to maximum treatment tumour volumes ≤10 cm^3^ and maximum dose based on body weight of <0.25 mg/kg (24, 25). Intratumoural TT elicits a rapid but localised inflammatory response, recruitment of immune cells, loss of tumour vasculature integrity, and induction of tumour cell death by oncosis (27).

In a multicentre, investigator- and owner-blinded study 120 dogs in the US were randomised into a treatment group (80 patients) and a control group (40 patients). Eligible MCT were not ulcerated or a recurrence from a previous surgical excision. Mast cell tumours were required to have a volume <10 cm^3^ and a minimum diameter >1 cm (≥1 cm diameter was required to use RECIST 1.1) (28). In addition, the calculated dose rate was required to be <0.25 mg TT/kg body weight. Seventy-five percent (60 out of 80) of dogs that received a single intratumoural TT treatment maintained a complete response (CR) in the target tumour to Day 84. A second intratumoural TT treatment for dogs that did not achieve a CR by Day 28 increased the CR rate to 88% (29). Longer term response durability was assessed for that study and at 12 months, 89% (57 out of 64) of patients that had a CR at Day 28 following a single treatment and were available for evaluation were still recurrence free at the treatment site (30).

This field study was designed as a safety and efficacy trial for the drug and pet owners were offered standard of care options for treatment before enrolment. Complete clinical staging was determined by the investigators with patients staged as Ia or IIIa (World Health Organisation staging criteria) at the time of screening based on absence of systemic signs of MCT metastasis using a combination of history, physical examination, systemic health assessment, and the absence of palpably enlarged locoregional lymph nodes (31). If enlarged lymph nodes were detected, a fine needle aspirate of the node was used to assess for potential metastasis. Finally, grading of the target MCT was determined via the Scarpa system of cytological grading (32). Dogs with all cytological grades of MCT were eligible for recruitment. The majority diagnosed were low grade MCT, with only nine diagnosed as HGMCT or suspect HGMCT, a prevalence broadly consistent with HGMCT occurrence in the general patient population (2).

While first-line standard of care for local MCT treatment is surgery and if clean margins are not possible, the addition of radiation therapy, chemotherapy, or both are often recommended for best outcomes (4, 14, 16, 20, 33). These adjunctive therapies would also be recommended for those patients presenting with high grade tumours or poor prognostic factors (4, 7, 11). It must be observed that although referral for these treatments may be offered, many owners may choose to not pursue such therapy for several reasons including cost, required time commitment, the need to regularly travel to a geographically distant specialist centre, or their pet's prognosis. For these situations an opportunity exists for an efficacious alternative to surgery that may provide local palliation of the tumour or longer durability of local tumour control (30).

The primary aim of this retrospective cohort study was to assess the efficacy of TT as a local treatment for cytologically graded canine HGMCT or suspected HGMCT and the long-term durability of that response.

## Methods

Study records from the US field study and from Australian studies were scanned for patients diagnosed with single cytologically confirmed HGMCT or suspected HGMCT treated with TT (1 mg/mL in buffered 40% propylene glycol) between 2013 and 2019 (29). Eighteen dogs, nine from Australian studies and nine from the US study, were included. Patients in the US trial had tumours graded using the Scarpa system (IDEXX Laboratories) and Australia enrolled patient tumours were graded using the Camus method (Independent Veterinary Pathology) (2, 29, 32). All patient owners were offered and declined referral or alternative therapies, including further staging after obtaining a HGMCT grading on fine needle aspirate cytology. All enrolled patients followed a mandated concomitant medication regimen before and after TT administration (Table 1) to reduce the potential for degranulation reactions (1, 6, 29). On the day of treatment, the target tumour was measured with calipers, and tumour volume calculated using the modified ellipsoid method: *tumour volume* = 0.5 × *length*(*cm*) × *width*(*cm*) × *depth*(*cm*) (29, 34–36). The TT dose was based on tumour volume (0.5 mg TT/cm^3^ tumour volume) and delivered intratumourally using a Luer lock syringe and a fanning technique to maximise distribution throughout the tumour mass (22, 29). Patients were restrained by hand during the procedure, sedation was permitted if considered necessary by the treating veterinarian, and personal protective equipment worn during administration of the TT dose (24, 29).

**Table 1:** Dosing schedule for concomitant medications to minimise risk of degranulation reactions with Day 0 the day of treatment.

	**Day ^−^2**		**Day ^−^1**		**Day 0**		**Day 1**		**Day 2**		**Day 3**		**Day 4**		**Day 5**		**Day 6**		**Day 7**	
	**a.m**.	**p.m**.	**a.m**.	**p.m**.	**a.m**.	**p.m**.	**a.m**.	**p.m**.	**a.m**.	**p.m**.	**a.m**.	**p.m**.	**a.m**.	**p.m**.	**a.m**.	**p.m**.	**a.m**.	**p.m**.	**a.m**.	**p.m**.
**CS^Ɨ^**	✓	✓	✓	✓	✓	✓	✓	✓	✓	✓	✓	✓	✓	✓	✓		✓		✓	
$H_{1}^{‡}$					✓	✓	✓	✓	✓	✓	✓	✓	✓	✓	✓	✓	✓	✓	✓	✓
$H_{1}^{\wedge }$					✓	✓	✓	✓	✓	✓	✓	✓	✓	✓	✓	✓	✓	✓	✓	✓

Ɨ *Corticosteroid: prednisolone/ prednisone 0.5 mg/kg PO bid*.

*^‡^H_1_ blocker: diphenhydramine 2 mg/kg PO bid [in Australian studies the H_1_ blocker used was chlorpheniramine (0.25–0.5 mg/kg)]*.

*^∧^H_2_ blocker: famotidine 0.5 mg/kg PO bid*.

Although this evaluation is retrospective in nature, these patients were assessed at regular intervals as part of larger cohort studies on Day 1, 7, 14, 28, and 84 for treatment response assessment and adverse event recording (37). Efficacy was assessed for each treated tumour at days 28 and 84 using response evaluation criteria in solid tumours (RECIST 1.1) and adverse events classified using the veterinary cooperative oncology group—common terminology criteria for adverse events (28, 37). For this study, responses were classified as either a CR or grouped as a not complete response (not-CR) if the patient response was classified as a partial response, progressive disease or stable disease. For dogs classified as not-CR after 28 days, the protocol was repeated with a second intratumoural TT injection. After the Day 84 treatment response assessment, long-term follow-ups were conducted at 6, 12, 18, and 24 months to assess presence or absence of a treatment site recurrence (TSR) with local recurrence defined as development of a cytologically confirmed MCT at or within 2 cm of the original TT treatment site (38–40). In the event of a TSR, patients involved in the Australian studies were reassessed, and if eligibility criteria were met, a repeat treatment cycle was administered. US patients could receive a single retreatment if on Day 28 a CR had not been achieved. If a patient in the US study was in the control group or diagnosed after Day 28 as not-CR, or with a TSR, they were ineligible for further treatment as part of that study (29).

## Results

Patient demographics of the 18 dogs included in this study are summarised in Table 2. Larger breed or mixed breed dogs were over-represented with Staffordshire Bull Terriers making up over a quarter of the group and no other breed had more than one individual.

**Table 2:** Summary of patient characteristics.

Median age in years on Day 0 (range)	8.5 (2–15)
Number of patients of each sex	
Female neutered (%)	9 (50)
Male neutered (%)	9 (50)
Breed	
Staffordshire bull terrier (%)	5 (28)
Median weight, kg (range)	18.7 (9.0–43.7)
Number of patients 9 ≤ 18 kg (%)	7 (39)
Number of patients >18 kg (%)	11 (61)

**Figure 1:**
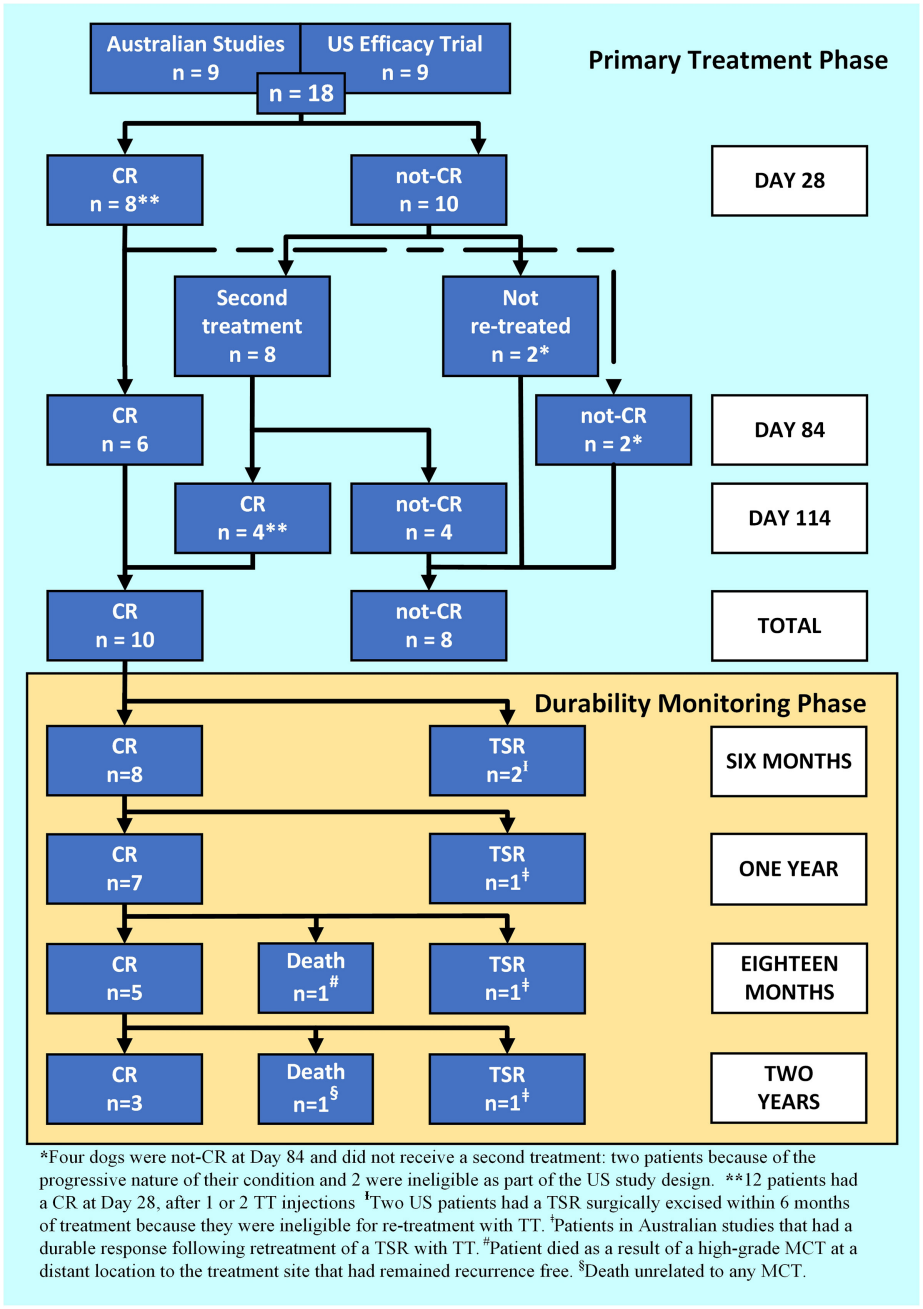
A patient response flowchart illustrating the Primary Treatment Phase response 84 days after Day 0 for 1, or 114 days for 2 tigilanol tiglate injections, and the Durability Monitoring Phase.

On Day 28, a CR was recorded in 44 % (8 out of 18) of patients after a single TT injection. Eight patients that did not achieve a CR with the first injection received a second injection. On Day 28, a CR was recorded in 67 % (12 out of 18) of patients after one or two injections. Eighty-three percent (10 out of 12) of patients maintained a CR to Day 84 (Figure 1: primary treatment phase). Eight patients were not-CR at Day 84 and of those, seven developed progressive disease and four subsequently died as a direct result within four months of treatment. Of the patients that died, two were classified as suspected HGMCT and two confirmed HGMCT. The remaining patient (US study) was recorded as a CR at Day 28 and not-CR at Day 84. This was likely a partial response on Day 28 and would have been a candidate for retreatment later but was ineligible because of the study design.

The median HGMCT volume on Day 0 (first treatment) was 1.1 cm^3^ (range 0.2, 10.4) and suspected HGMCT volume was 2.6 cm^3^ (range 0.3, 3.8). The corresponding number and percentage of CRs for each tumour location, volume, and tissue layer is summarised in Table 3. Seventy percent (7 out of 10) of smaller volume (≤2 cm^3^) confirmed or suspected HGMCT had a CR. Thirty-eight percent (3 out of 8) of larger volume (>2 cm^3^) had a CR, and all three were classified as suspected HGMCT.

**Table 3:** Summary of cytological grade classification with number of patients and complete responses for each tumour location, volume, and tissue layer 84 days after 1, or 114 days for 2 TT treatments.

	**Cytological grade classification**			
	**Confirmed high**		Suspected high^*^	
	**No. of cases** **(% of total)**	**No. of CRs** **(% of group)**	**No. of cases** **(% of total)**	**No of CRs** **(% of group)**
**Tumour location**				
Trunk (%)	4 (22)	2 (50)	2 (11)	1 (50)
Limb (%)	4 (22)	2 (50)	3 (17)	2 (67)
Genital/Perianal (%)	2 (11)	1 (50)	1 (6)	1 (100)
Head (%)	1 (6)	1 (0)	1 (6)	1 (100)
**Tumour volume on Day 0 (cm** ^**3**^ **)**				
≤ 2 (%)	7 (39)	5 (71)	3 (17)	2 (67)
2-10.4 (%)	4 (22)	0 (0)	4 (22)	3 (75)
**Tissue layer^Ɨ^**				
Cutaneous (%)	9 (50)	3 (33)	5 (28)	5 (100)
Subcutaneous (%)	2 (11)	2 (100)	2 (11)	0 (0)
**Total number (%)**	**11 (61)**	**5 (45)**	**7 (39)**	**5 (71)**

* *Inclusive of 1 sample classified likely and 1 sample classified as possible*.

Ɨ *Tissue layer was visually assessed and not confirmed by surgical biopsy*.

Of the 18 patients in this cohort, five patients recorded 15 adverse events that were graded 3 or 4 (37). One patient had a degranulation reaction, as a result of not receiving concomitant medications and two patients experienced significant pain requiring additional analgesia. Lameness was an expected adverse event, but two experienced a higher severity during this study. A further patient developed a large tissue deficit (1 of the 2 two largest in the US pivotal study) that healed fully but was 1 of 3 (out of 117 patients) that took longer than 84 days to heal (29, 41).

For the 10 dogs that maintained a CR to 84 days after their last TT treatment, durability of the target tumour response was followed (Figure 1: durability monitoring phase, Table 4). At 1-year post-treatment 70% (7 out of 10) of patients that had a CR, maintained that response and were recurrence free. At 2 years post-treatment, 60% (6 out of 10) of patients were alive, 50% (3 out of 6) of those had remained recurrence free at the treatment site. The remaining three patients, enrolled in Australian studies, received a second treatment cycle and had at least a further 18 months recurrence free. Of the five patients that developed a TSR, two did within 6 months and both were US study participants not eligible for a second treatment cycle as part of the US study design. In addition, two of the TSRs were from cases that had been classified as suspected HGMCT (Table 4).

**Table 4:** Long-term treatment durability responses after high-grade and suspected HGMCT patients recorded a complete response 84 days after 1 or 2 TT treatments.

**Durability classification**	**Cytological grade**		**Total**
	**Confirmed high-grade**	**Suspected high-grade**	
1 year	4	3	7
2 years	1	2	3
TSR	3	2	5
Over 2 years with >1 treatment cycle	2	1	3

## Discussion

The aim of this retrospective cohort study was to assess the efficacy and long-term durability of TT as a local treatment for canine HGMCT. This study found that 56% (10 out of 18) of patients had a complete treatment response, after one or two TT injections and maintained that response beyond 84 days. Forty percent (4 out of 10) of patients achieving a CR required 2 injections in the primary treatment phase. The Day 28 response rate to the first injection (8 out of 18, 44%) was comparable to the second injection (4 out of 8, 50%). Likewise, the utilisation of repeat treatment cycles to prolong response durability in the event of a TSR was used to beneficial effect. At 2 years, three out of six patients that were alive and available for evaluation had required a second treatment cycle and all had a further tumour free interval >18 months.

High-grade mast cell tumours have a more aggressive biological behaviour that warrants an appropriate treatment approach and guarded expectations of patient prognosis. Metastatic rates for HGMCT or undifferentiated tumours are reported to be over 50% with most dogs dying within a year of diagnosis (11, 42). Numerous clinical reports have shown improved survival with multimodality treatment including surgery or radiation therapy for locoregional control and chemotherapy (12–14, 16, 17, 19). Studies have also suggested that locoregional control may be more important regarding improving progression free survival times in dogs with HGMCT (14, 16, 19). These studies have demonstrated survival times ranging between 1 and 2 years with locoregional surgery or irradiation. It is not surprising that the patients in this current study that did not respond to TT treatment developed progressive disease and 4 died from complications related to their MCT disease within 4 months. This is consistent with median survival times of HGMCT patients reported in other studies (11).

Our study demonstrated a potential relationship between response rate and tumour volume. The smaller volume (≤2 cm^3^) HGMCT had a response rate comparable to other studies, but the larger volume (>2 cm^3^) had a poorer response rate (29). Tumour volume has been confirmed as a prognostic factor for HGMCT in other studies (14, 16). This relationship has not been found with TT treatment across much larger sample sizes when treating low grade MCT (29, 41). Owing to the small retrospective cohort size there were too few larger volume HGMCT cases for significant comparison and the *post-hoc* nature of any statistical analysis regarding efficacy of subgroups would be inaccurate (43).

The creation of a tissue deficit appropriate to tumour volume is an indicator of efficacy (29, 41). In the event of a perceived incomplete tumour response the treatment interval should not be <28 days and any tissue deficit should be allowed to heal before retreatment. In most cases, this allows for the true extent of any residual tumour to be assessed, confirmed, measured, and treated. This study showed a benefit to repeat doses of TT with an increase in CR rate from 44 to 56% with two injections with local tumour control leading to improved survival. A new treatment cycle should be considered for any subsequent TSR. In Australian studies there has been no detection of reduced efficacy related to the number of treatment cycles on a location or the subsequent use on *de novo* tumours arising at a distant location. There is a low likelihood of the development of resistance to TT given the host's immune response plays a role in TT mode of action and may improve patient survival (27, 44, 45). The beneficial effect of the combination of TT for local tumour control with adjunctive systemic chemotherapy protocols or tyrosine kinase therapy is unknown and prospective studies are necessary to provide the answer.

The population characteristics of this small cohort of patients were consistent with other larger studies. The median age was 8.5 years, all the patients had been neutered and there was an even distribution of gender. There was an overrepresentation of medium to large dogs with Staffordshire Bull Terriers featuring prominently (46–49). The number of patients in this study was limited by its retrospective nature, and the small sample size was too low for any definitive statistical analysis. The US clinical study evaluated the efficacy of a single intratumoural dose of TT compared to a control, and strategies to improve the treatment efficacy of HGMCT was beyond the scope of that study. We recognize the repeat treatment of selected US study participants may have improved overall treatment efficacy or enabled prolonged durability in four out of nine US patients.

There are several weaknesses with this study, with regard to its retrospective nature, including the small cohort of patients, the use of cytologic grading and inconsistent staging. Histologic grading criteria is perhaps the most often used prognostic factor to predict MCT biological behaviour and a patient's prognostic outcome. As with any single diagnostic test, Patniak (1984) and Kiupel (2011) methods of grading have limitations regarding interobserver agreement and clinical outcomes (50). These methods also require incisional tissue biopsy or excision of the tumour for grading assessment. Although more comprehensive validation of cytological grading is required, this method is better suited to an intratumoural approach. A tissue biopsy results in damage to the surface integrity of a tumour and is likely to result in drug leakage at a biopsy site. Cytological evaluation has the added benefits of being less invasive, less expensive, and not requiring anaesthesia for sample collection.

Fine needle aspirate samples were taken from all the MCT in this study and graded using either the Scarpa (US study) or Camus (Australian studies) cytological 2-tier system (2, 32). The Camus (2016) 2-tier cytological system has 88% sensitivity and 94% specificity when compared to Kiupel, but with a positive predictive value of 68% there is a tendency to overestimate the number of HGMCT. False positives (up to 32% of HGMCT cases) which in turn may increase the overall reported survival times (2, 3, 11). Whilst a cytological 2-tier (high or low) grading system was followed, 39% (7 out of 18) of samples were classified as suspected high-grade with four out of the seven from Australian studies. These patients were included in this study and the distinction of those classified as confirmed and suspected HGMCT is clearly reported in the Results section (Tables 3, 4). The CR rate of confirmed HGMCT was 45% (5 out of 11) compared to 71% (5 out of 7) in suspected HGMCT. In addition, four MCT in this study were classified as subcutaneous HGMCT by investigators based on palpation. These were not confirmed as subcutaneous by biopsy as is standard for accurate differentiation (42). Efforts have been made to utilize grading schemes for subcutaneous MCT and not surprisingly, there continues to be controversy in its accuracy and use for the subcutaneous variants. A recent study evaluated the pathology of subcutaneous MCT to predict prognosis. Similar to the Camus and Scarpa cytological grading systems, this study found mitotic index and presence of multinucleation linked to poorer prognosis (11, 32, 42). In addition, repeat testing of samples classified as suspected HGMCT may have clarified the cytologic grade and reduced ambiguity when assessing the results of this study.

Routine diagnostic staging was often not completed as the pet owners had elected no further treatment, assuming a poor prognosis. Despite the lack of consistent staging, several of these patients remained tumour free and exhibited long survival times and inconsistencies of this nature have been described in other retrospective studies (39). A more recent evaluation of patients with HGMCT with clinical stage 1 disease treated with combination therapy reported a median survival time of 1,046 days with 1 and 2-year survival rates of 79.3 and 72.9% (39). In retrospect, under-staging patients with the assumption of no metastasis would decrease survival times due to potential recruitment of patients with higher stages of disease at the time of diagnosis (39).

Tigilanol tiglate was chosen as a method of local tumour control in this subgroup with the intent to improve patient quality of life or prevent its decline due to local tumour progression. As a result, adverse events were experienced that were consistent and expected, in line with TT's mode of action. At first glance, the creation of a wound secondary to tumour necrosis may seem counterintuitive toward improved quality of life, however within 2 weeks of TT treatment there were no reports of deterioration by the owners or the investigating veterinarians. Owners of dogs receiving TT considered their dog's health to have improved compared to owners of control dogs that had not at that point received treatment since the initial diagnosis (29).

This cohort of HGMCT cases highlights that TT has efficacy as a local treatment for this grade of tumour but suggests that it is more efficacious for smaller volume (≤2 cm^3^) tumours. Larger diameter HGMCT have been found to have reduced median survival times in other studies (14, 16, 39). This is not the case for low grade MCT, where lesions up to 10 cm^3^ in volume can be treated efficaciously with TT (29). Lower recurrence rates have been observed in Australian studies with low grade MCT, but the same principal of retreatment can be applied (30, 51). A retreatment strategy, wherever a TSR is diagnosed, after 28 days should be implemented to prolong response durability and this should be a straight-forward consideration if pre-treatment criteria are still met. Tigilanol tiglate provides an alternative treatment approach to local treatment in patients with cytologically diagnosed HGMCT that would either pose an unacceptable anaesthetic risk, the tumour location provides a challenge when attempting surgical excision, or where the pet owner refuses referral for combination therapy and opts for palliative care.

## Data Availability Statement

The raw data supporting the conclusions of this article will be made available by the authors, without undue reservation.

## Ethics Statement

The animal study was reviewed and approved by Queensland Department of Agriculture and Fisheries, Community Animal Ethics Committee (Australian participants). Institutional animal ethics was not required for US participants as the study was under a USFDA Protocol—Investigational New Animal Drug (No. 1-012436, July 25, 2016). Written informed consent was obtained from the owners for the participation of their animals in this study.

## Author Contributions

GB was responsible for data compilation and manuscript preparation. GB and JC performed components of the clinical work. PJ, JC, TD, PR, and CJ reviewed the manuscript. All authors contributed to the article and approved the submitted version.

## Conflict of Interest

GB, JC, PJ, TD, and PR are employed by QBiotics Group Limited and the remaining author, CJ, receives payment by them as an independent consultant. This work was funded by QBiotics and they own the intellectual property and patents associated with tigilanol tiglate.

## Publisher's Note

All claims expressed in this article are solely those of the authors and do not necessarily represent those of their affiliated organizations, or those of the publisher, the editors and the reviewers. Any product that may be evaluated in this article, or claim that may be made by its manufacturer, is not guaranteed or endorsed by the publisher.
